# G-Quadruplexes in the Viral Genome: Unlocking Targets for Therapeutic Interventions and Antiviral Strategies

**DOI:** 10.3390/v15112216

**Published:** 2023-11-05

**Authors:** Rajiv Pathak

**Affiliations:** Department of Genetics, Albert Einstein College of Medicine, New York, NY 10461, USA; rajivpathak17@gmail.com

**Keywords:** G-quadruplex, G-quadruplex forming sequences, non-canonical G4s, virus, targeting G4s, G-quadruplex ligands, small-molecule drugs, G4-interacting proteins, oligonucleotides, antiviral activity

## Abstract

G-quadruplexes (G4s) are unique non-canonical four-stranded nucleic acid secondary structures formed by guanine-rich DNA or RNA sequences. Sequences with the potential to form quadruplex motifs (pG4s) are prevalent throughout the genomes of all organisms, spanning from prokaryotes to eukaryotes, and are enriched within regions of biological significance. In the past few years, the identification of pG4s within most of the Baltimore group viruses has attracted increasing attention due to their occurrence in regulatory regions of the genome and the subsequent implications for regulating critical stages of viral life cycles. In this context, the employment of specific G4 ligands has aided in comprehending the intricate G4-mediated regulatory mechanisms in the viral life cycle, showcasing the potential of targeting viral G4s as a novel antiviral strategy. This review offers a thorough update on the literature concerning G4s in viruses, including their identification and functional significance across most of the human-infecting viruses. Furthermore, it delves into potential therapeutic avenues targeting G4s, encompassing various G4-binding ligands, G4-interacting proteins, and oligonucleotide-based strategies. Finally, the article highlights both progress and challenges in the field, providing valuable insights into leveraging this unusual nucleic acid structure for therapeutic purposes.

## 1. Introduction

**Figure 1:**
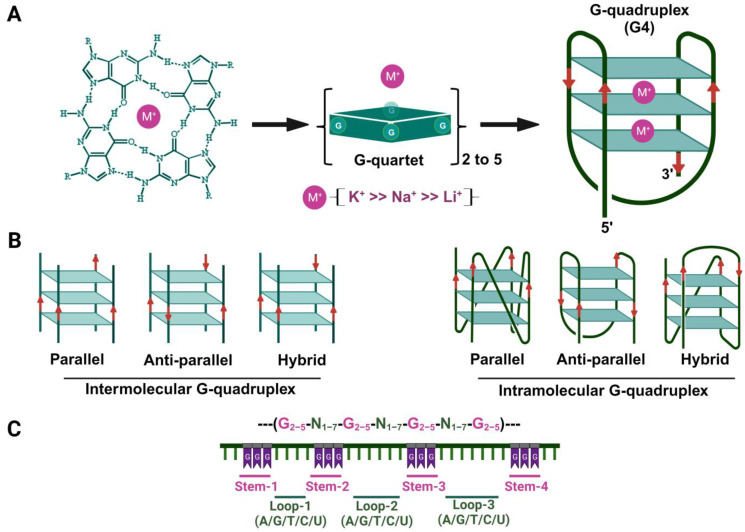
Building blocks and structures of the G-quadruplex (G4) and its various topologies. (**A**) Chemical structure of four guanines linked together through eight Hoogsteen hydrogen bonds (left panel) and a schematic illustration of a planar guanine tetrad (middle panel) as well as a G-quadruplex stabilized with monovalent cations (M^+^) (right panel). Red arrows in the backbone denote 5′-to-3′ strand direction; (**B**) schematic representation showing different topologies of intermolecular (left panel) and intramolecular G4 structures (right panel); (**C**) the conventional nucleotide sequences capable of forming a G-quadruplex structure, where “N” represents the loop sequences (created with BioRender.com).

G-quadruplexes (G4s) are four-stranded DNA or RNA secondary structures composed of guanine-rich sequences [1,2]. These structures consist of stacked G-quartets held together by Hoogsteen hydrogen bonding and further stabilized by charge coordination with several monovalent and divalent cations under physiological conditions (Figure 1A) [1]. G4 structures are highly polymorphic in nature whose topology depends on variations in strand stoichiometry, polarity, loop characteristics, and their positional arrangement within the sequence. G4 structures can be classified as intramolecular or intermolecular, while their topologies can be categorized as parallel, antiparallel, or hybrid, depending on the direction of the strands (Figure 1B) [1]. Over the years, with the advancement of sequencing technologies, the number of sequencing data from various organisms has dramatically increased. As a result, the creation of databases and algorithms has surged, empowering the exploration and mapping of potential G-quadruplex forming sequences (PQSs) within the genomes of all organisms, ranging from prokaryotes to eukaryotes [3,4,5,6]. The complete sequencing of the human genome unveiled approximately 300,000 unique locations with the potential to form G4 structures [7]. Extensive research has demonstrated that potential quadruplex (pG4) motifs occur within diverse biologically significant genomic regions, such as telomeres, promoters, and introns. These motifs play significant roles in crucial biological processes, including DNA replication, transcription, recombination, epigenetic regulation, and the maintenance of telomere stability. The presence of pG4 motifs may regulate gene expression by either modulating specific gene expression mechanisms or by altering the recruitment of specific transcription factors and co-factors. Additionally, G4 structures can impede transcriptional elongation either directly or indirectly, potentially acting as roadblocks in the process. In addition to humans, PQSs have also been identified in the genomes of other organisms, such as mammals [3,6], yeasts [8], protozoa [9], and bacteria [4,5]. Remarkably, these motifs serve as building blocks of gene regulatory networks and exhibit evolutionary conservation across various stages of evolution [5,10,11]. The existence and functional significance of these pG4 motifs underscores their crucial role in shaping genetic regulatory mechanisms across a wide range of organisms.

Over the years, there has been a growing fascination with G4s, leading to numerous publications exploring their biophysical and chemical attributes along with biological functions in both prokaryotes and eukaryotes. Despite this surge in interest and extensive research, the potential physiological role of G4s has been a subject of ongoing debate for an extended period. The research community devoted to G4s has been diligently attempting to uncover cellular evidence for the direct visualization of such non-B-form DNA structures within live cells. Direct evidence for the in vivo formation of G4 motifs was first established in the lower eukaryote *Stylonychia lemnae*, where the presence of these structures at the telomeric ends of the organism was identified through Immunofluorescence techniques [12]. Consequently, the detection of G4 loops within the bacterial system was reported for the first time using electron microscopy when human G-rich DNA sequences were transcribed in *E. coli* [13]. To date, the most compelling evidence for the existence of these unconventional structures has gained substantial support from innovative visualization approaches that employ G4-specific antibodies and chemical probes [14,15,16,17]. In a subsequent experiment involving live-cell single-molecule fluorescence imaging, a G4-specific fluorescent probe (SiR-PyPDS) was used to specifically target G4 structures. This probe enabled the real-time detection of individual G4 structures at single-molecule levels, revealing their presence within living cells. The study also unveiled that the formation of G4 structures is cell-cycle-dependent and undergoes dynamic transitions between folded and unfolded states within live cells [18]. These discoveries not only provide substantial evidence for the existence of G4-mediated regulatory networks within cells but also underscore the potential of G4 structures as promising targets for therapeutic interventions.

The significance of G4-forming sequences as regulatory elements is evident not just in prokaryotic and eukaryotic cells but also in numerous Baltimore groups of viruses, including those responsible for recent epidemics like Ebola, and SARS-CoV-2 [19,20,21,22,23,24,25]. While viral genomes display diversity, PQSs demonstrate remarkable conservation within individual viral species. This implies a crucial role of G4s in viral evolution, potentially influencing important aspects of the viral life cycle and adaptation over time [25]. Over the past few years, the identification of G4s within viruses has garnered substantial attention, mainly due to their presence in regulatory regions of the viral genomes. In this context, the utilization of specific G4 ligands has facilitated the elucidation of intricate G4-mediated regulation occurring during critical stages of viral life cycles. This exploration has not only enhanced our understanding of G4-related mechanisms but has also unveiled the potential of targeting viral G4s as a promising avenue for novel antiviral approaches. As a result, scientists worldwide are intrigued by the therapeutic potential offered by small molecules capable of either stabilizing or disrupting G4 formation. Multiple reports outline the utilization of G4-interacting compounds that exhibit antiviral activity within infected cells, thereby implicating G4s in numerous human viral diseases. Taken together, the presence of PQSs underscores their potential significance in viruses and offers new avenues for comprehending and managing viral infections.

This article aims to provide a comprehensive overview of the existing literature concerning the identification, formation, and functional relevance of G4 structures in human-infecting viral genomes. Additionally, it emphasizes various G4-targeting strategies, such as G4-binding ligands, G4-associated proteins, and G4-based oligonucleotides, all of which demonstrate antiviral effects. Furthermore, the article addresses the current advancements, challenges, and future prospects in utilizing G4s for the development of antiviral interventions.

## 2. Bioinformatic Prediction of Potential G-Quadruplex-Forming Sequences (PQSs or pG4)

As sequencing data expands, databases and algorithms have also emerged to facilitate the search and mapping of PQSs in several mammalians and bacterial species. The typical sequence forming G-quadruplexes is generally depicted as -G_(≥2)_-N_1–7_-G_(≥2)_-N_1–7_-G_(≥2)_-N_1–7_-G_(≥2)_-*,* with groups of two to five guanosines interspersed by one to seven nucleotides, which can represent any nucleotide (Figure 1C). While exceptions exist where N can exceed seven, longer loop lengths introduce flexibility and destabilize the G4 structure [26]. Several algorithms have been developed to evaluate the likelihood of a sequence forming G4s and to analyze the distribution of these PQSs across various genomes. In this regard, multiple computational tools, such as QGRS Mapper, QuadBase, QuadBase2, Quadparser, PQSFinder, G4Hunter, and G4-iM Grinder, have been developed to predict and identify PQSs in various prokaryotic and eukaryotic genomes [27,28,29,30,31,32]. The rapid advancement and optimization of bioinformatic tools for detecting G4 motifs has greatly sped up the discovery of PQSs in viruses. Notably, many of these identified sequences have indeed been shown to adopt G4 structures under in vitro conditions [23]. Nevertheless, it’s important to exercise caution and not automatically presume G4 formation from predicted PQSs, as not all PQSs have been shown to fold into a G4 structure experimentally.

## 3. Presence and Functional Role of Potential G-Quadruplexes in Viruses

**Figure 2:**
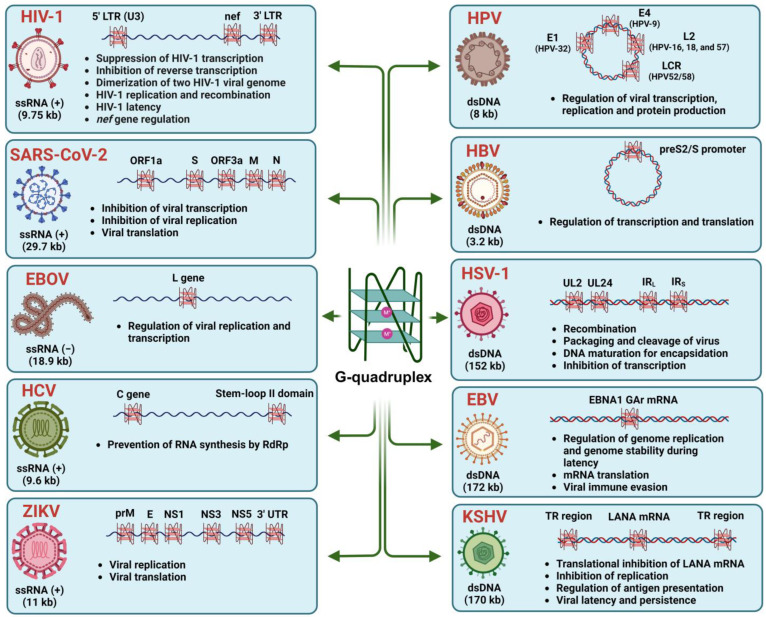
Schematic representation summarizing the presence and functional relevance of G-quadruplexes in viruses. Each virus is depicted with its virion structure, genome organization, genome size, a schematic representation of G4 locations in the viral genome, and the functional roles of G4s in the viruses (created with BioRender.com).

While the concept of DNA G4s was first introduced by Gellert et al. in 1962 [33], the initial indication of G4 formation in viral genomic RNA was proposed by Sundquist et al. in 1993 [34]. Since then, studies on G4s within viruses have flourished, gaining significance across various systems and expanding the scope of research in this area. Furthermore, the development of bioinformatic tools for detecting G4 motifs has significantly expedited the identification of PQSs in viruses. These pG4 motifs have been found to be enriched and conserved in crucial positions within the viral genome across all seven classes of viruses in the Baltimore classification system, including ssDNA, dsDNA, dsRNA, negative-sense ssRNA, positive-sense ssRNA, positive-sense ssRNA reverse transcriptase viruses, and double-stranded DNA reverse transcriptase viruses (Table 1) [19,20,25,35]. PQS presence has been observed in various viruses across different families, such as Herpesviridae and Papillomaviridae (dsDNA viruses) [36,37,38,39,40,41,42], Flaviviridae and Filoviridae (ssRNA+ viruses), Retroviridae (RNA viruses with reverse transcriptase), and Hepadnaviridae (dsDNA viruses with reverse transcriptase) (Table 1) [21,25,43]. While the viral genomes may differ, the occurrence and location of PQSs remain relatively conserved within specific virus classes and families, implying a distinct biological role for G4s throughout evolution [25]. This article discusses the presence and functional relevance of G4s in specific viruses, where their existence has been verified (Figure 2).

### 3.1. Human Immunodeficiency Virus Type 1 (HIV-1)

The human immunodeficiency virus-1 (HIV-1), responsible for acquired immunodeficiency syndrome (AIDS), is classified as a member of the Lentivirus genus in the Retroviridae family. The HIV-1 genome comprises two identical positive-sense single-stranded RNA molecules enclosed within the virus particle’s core along with the necessary enzyme machinery for viral replication [77]. After undergoing reverse transcription within the host cell’s cytoplasm, the newly synthesized dsDNA of HIV-1 is subsequently transported to the cell nucleus, where it becomes integrated into the host cell’s genetic material. The presence of G4 structures have been identified in both the RNA and DNA forms of the HIV-1 genome, playing a significant role throughout the virus’s life cycle including genome recognition, recombination, dimerization, and packaging of HIV-1 [19,44].

The HIV-1 long terminal repeat (LTR) plays a crucial role in regulating the transcription of viral genes and the entire genome within the integrated provirus. These proviruses contain two identical LTR sequences at both ends, comprising U3, R, and U5 regions. Notably, the U3 region of the 5’ LTR exhibits highly conserved G-rich sequences that correspond to the binding sites of Sp1 and NF-κB. Transcription initiation occurs when the U3 promoter sequence of LTRs interacts with various cellular factors, such as NF-κB, Sp1, and RNA polymerase II [78]. Among the twelve pG4 sequences identified in the U3 region, two G4 structures were identified, named LTR-II and LTR-III, which consist of three stacked G-quartets and can form stable intramolecular G4s [45]. Disrupting the formation of G4s through mutations increased the activity of the HIV-1 promoter in cells, while treatment with a G4 ligand hindered promoter activity [45]. Interestingly, one of the LTR G4s, named LTR-IV, did not form readily under physiological ionic conditions but could be induced by the presence of a G4 stabilizing ligand. The nuclear magnetic resonance (NMR) structure analysis of LTR-IV revealed the formation of a well-defined parallel-stranded G4 structure which includes a thymine bulge at its 3’-end [79]. Piekna-Przybylska et al. showed that the U3 region’s G-rich sequences consists of three specific binding elements that interact with the Sp1 transcription factor. These binding elements can adopt various forms of G4 structures, and the Sp1 protein is capable of recognizing and binding to its specific site when folded into a G-quadruplex [44]. The G-rich sequence found in the U3 region of the HIV-1 genome is not only present in the viral DNA but also in the HIV-1 RNA genome. Furthermore, Piekna-Przybylska et al. demonstrated that the RNA sequence of U3 has the ability to form dimers, exhibiting characteristics similar to intermolecular G4s. These findings, coupled with previous studies demonstrating G4 dimers in the gag and cPPT regions, indicate that multiple intermolecular G4s formed at different locations in the RNA genome contribute to maintaining the integrity of the two viral genomes [44].

Another separate study revealed that the cellular protein nucleolin has the ability to specifically recognize and stabilize G4 structures found in the LTR promoter resulting in enhanced suppression of HIV-1 transcription. Conversely, when nucleolin binding was disrupted in cells, there was a significant increase in LTR promoter activity. These findings demonstrate that nucleolin has a specific role in the G4-mediated regulation of the HIV-1 LTR promoter [80]. On the other hand, the human ribonucleoprotein (hnRNP) A2/B1 was identified as another protein interacting with LTR G-quadruplexes, acting as a HIV-1 transcription activator [81].

Furthermore, it has been observed that a specific segment of the reverse-transcribed pre-integration HIV-1 genome, known as the central DNA flap, can form an intermolecular parallel DNA quadruplex. This specific DNA quadruplex interacts with the HIV-1 nucleocapsid protein (NCp), providing protection to the pre-integrated genome against degradation by nucleases [82]. The HIV-1 NCp in turn facilitates the unfolding of the monomeric G-quartet while stabilizing the dimeric interaction. The presence of these G4-forming sequences corresponds to recombination hot spots in the gag region and demonstrates an increased rate of template switching, suggesting a potential role for these structures in HIV-1 replication and recombination processes [83]. Further studies have provided evidence indicating that the dimerization of HIV-1 is facilitated by the formation of RNA G4s, which play a significant role in various stages of the virus’s life cycle [34]. Specifically, the nucleocapsid protein- NCp7 of HIV-1, which remains bound to the viral RNA during reverse transcription, is responsible for unraveling stable RNA G4s present in the U3 Region of the HIV-1 RNA genome. This unfolding process promotes the formation of DNA or RNA duplexes, enabling the progression of reverse transcription [84].

Nef, another HIV-1 accessory protein plays a crucial role in proviral DNA synthesis and the establishment of persistent infection [85]. It is encoded in the 3’-end of the viral genome and partially overlaps with the 3’-LTR. By conducting a comprehensive computational analysis of PQSs within the HIV-1 *nef* coding region, researchers identified three conserved sequences capable of forming G4 structures. These G4 structures were found to be highly stabilized by G4-specific ligands and might naturally function as a molecular switch, controlling the viral protein expression [46]. The evidence presented here highlights the presence and regulatory role of G4s in the HIV-1 life cycle. Furthermore, it suggests that targeting G4s using specific proteins or ligands could potentially interfere with HIV-1 infection. This implies that G4s could serve as potential targets for the development of novel therapeutic strategies against HIV-1.

### 3.2. Severe Acute Respiratory Syndrome Coronavirus-2 (SARS-CoV-2)

Coronaviruses are enveloped, single-stranded positive-sense RNA viruses that belong to the Betacoronavirus genus within the Coronaviridae family. As of yet, there have been seven strains of coronaviruses known to infect humans, including the global outbreak of severe acute respiratory syndrome coronavirus 2 (SARS-CoV-2), the virus responsible for the COVID-19 pandemic. COVID-19, a viral respiratory disease, emerged in late 2019 in Wuhan, China, and quickly spread across the globe, causing widespread devastation [24]. In the last couple of years, multiple studies have identified the presence of potential G-quadruplex structures in the genome of SARS-CoV-2 [50,86,87,88,89,90].

Panera et al. used the QGRS Mapper to explore PQSs in the SARS-CoV-2 genome. They applied the motif G_x_N_y_G_x_N_y_G_x_N_y_G_x_, with x indicating the number of G tetrads (≥2) and y representing the loop length (from 0 to 36). The analysis, with specific criteria, revealed 25 potential G4 structures in the SARS-CoV-2 genome [86]. Like the above findings, Ji et al. also utilized the QGRS Mapper and not only discovered 25 four contiguous GG runs (G_2_N_x_G_2_N_y_G_2_N_z_G_2_) in the SARS-CoV-2 genome but also observed that some PQSs were conserved within the Coronaviridae family. The G4 motifs were primarily located in the open reading frame regions of ORF1ab, spike (S), ORF3a, membrane (M), and nucleocapsid (N) genes. However, PQSs containing four contiguous GGG runs or GGGG runs were not found in the SARS-CoV-2 genome [87]. Among the 14 highly conserved PQSs across various coronaviruses, two top-ranked PQSs at positions 13,385 (ORF1a) and 24,268 (S gene) were experimentally shown to form RNA G4 structures using multiple spectroscopic assays. Additionally, these structures were found to interact directly with the viral helicase (Nsp13), as confirmed via microscale thermophoresis. Molecular docking models suggest that Nsp13 induces distortion in the G4 structure by causing the guanine bases to flip away from the guanine quartet planes [87]. By utilizing the QGRS mapper and QuadBase2, Cui et al. made significant predictions, revealing that all seven human coronaviruses harbor pG4 motifs. These sequences were identified in the open reading frame regions of ORF1ab, S, and N genes, which were further verified by circular dichroism (CD) spectroscopy and Thioflavin T fluorescence assay [50]. Notably, four coronaviruses—including SARS-CoV, SARS-CoV-2, HCoV-OC43, and HCoV-229E—shared conserved G4 sequences in the ORF1ab region. Additionally, five coronaviruses, encompassing SARS-CoV, SARS-CoV-2, MERS-CoV, HCoV-NL63, and HCoV-229E, shared conserved G4 sequences in the S protein-coding region. Furthermore, Cui et al. identified a potential association between SARS-CoV-2 Nsp3 and G-quadruplex sequences, like the previously discovered SARS-CoV Nsp3 protein by Kusov et al., which is implicated in unwinding G4s in RNA and contains two SUD (M and N) domains capable of interacting with G4 sequences [50,91]. In their study, Belmonte-Reche et al. utilized an upgraded version of the open-source algorithm, G4-iM Grinder, to analyze the SARS-CoV-2 reference genome. Through this analysis, they identified 71 PQSs with a medium probability score ranging from 20 to 40. Comparing the results to previous reports on quadruplex-related analysis in the single strand of SARS-CoV-2, G4-iM Grinder revealed 47 additional PQSs that had not been previously reported for the SARS-CoV-2 but exhibited the same probability of forming G4s. This finding highlights the utility of G4-iM Grinder in expanding our understanding of pG4 motifs in the SARS-CoV-2 genome [88]. In another study, by employing a combination of bioinformatic and biophysical methods, Kabbara et al. were able to further detect 16 highly conserved sequences that were most likely to form G4 structures. In the identified G4 structures, 11 sequences exhibit multi-molecular G4s, while 5 sequences display mono-molecular G4s. Notably, mono-molecular G4s are predominantly found in the ORF1a gene of SARS-CoV, SARS-CoV-2, and MERS-CoV as well as in the S genes of SARS-1 and MERS [92].

Considering the variation in G4 predictions arising from different software and algorithms, it is crucial to validate these findings using in vitro methods like CD spectroscopy and NMR as well as in vivo experiments such as targeted ligand capture. It is worth noting that PQSs located at various positions, including 13,385, 24,268, and 28,903, among others, have already been validated using different biophysical techniques [50,51,86,88,93]. In their research, Zhao et al. employed QGRS Mapper and G4 RNA screener to analyze the SARS-CoV-2 genome, leading to the identification of four PQSs, named RG-1, RG-2, RG-3, and RG-4. Notably, RG-1, situated in the coding sequence region of the SARS-CoV-2 nucleocapsid phosphoprotein (N), was experimentally confirmed to form a stable RNA G4 structure in live cells. This finding represents the first concrete evidence that PQSs within the SARS-CoV-2 genome can indeed form G4 structures within living cells [51].

### 3.3. Herpesviruses

Herpesviridae, a large virus family, possesses a linear double-stranded DNA genome ranging from 125–235 kb in size. These viruses are extensively studied and exhibit a broad host range, infecting various organisms. Among the members of the Herpesviridae family, at least eight distinct species are known to infect humans. The Herpesviridae family is classified into three subfamilies based on biological features and genomic attributes: Alphaherpesvirinae, Betaherpesvirinae, and Gammaherpesvirinae. The Alphaherpesvirinae subfamily comprises important human herpesviruses (HHVs) such as HSV-1 (HHV-1), HSV-2 (HHV-2), and Varicella-zoster virus (HHV-3). The Betaherpesvirinae subfamily includes human cytomegalovirus (HCMV or HHV-5), human herpesvirus 6 (HHV-6A and HHV-6B), and human herpesvirus 7 (HHV-7). The Gammaherpesvirinae subfamily consists of the remaining HHVs, including Epstein–Barr virus (EBV or HHV-4) and Kaposi’s-sarcoma-associated herpesvirus (KSHV or HHV-8) [94]. One of the first systematic genome-wide bioinformatic analysis of G4s in herpesviruses revealed an exceptionally high density of PQSs across all species. The study by Biswas et al. revealed PQS densities as high as 1.037 per kilobase (kb) among herpesviruses, which is more than seven times higher than the density observed in the human genome. The human herpesvirus genomes contained an average of 14 to 318 PQS, with HHV-2 having the highest number of PQS (*n* = 318) among all human herpesviruses [67]. The subsequent section explores certain herpesviruses in detail with respect to the presence of PQSs.

#### 3.3.1. Herpes Simplex Virus Type 1 (HSV-1)

HSV-1 causes lifelong persistent infections characterized by latency and reactivation/lytic replication cycles. HSV infections affect over half of the global population and can lead to severe outcomes in immunocompromised individuals. Through an extensive genome-wide bioinformatic analysis, nine regions in the HSV-1 genome were identified as having highly repeated potential QGRS (quadruplex-forming G-rich sequences). Among these regions, six were located in the leading strand of the *gp054* gene responsible for encoding UL36, the largest viral protein. CD spectroscopy confirmed that all nine putative QGRS fold into remarkably stable G4 conformations with various forms, including hybrid (*gp054*), antiparallel (*un2*), and parallel (*un1*, *un3*) [36]. The formation of G4 structures in HSV-1 infected cells were visualized using a G4-specific monoclonal antibody, demonstrating their formation and specific localization within the cells in a virus cycle-dependent manner [37].

#### 3.3.2. Epstein–Barr Virus (EBV or HHV-4)

Epstein–Barr virus (EBV) is a member of the Herpesviridae family and Lymphocryptovirus genus and is characterized by its double-stranded DNA structure. In addition to the commonly known infectious mononucleosis, EBV is linked to a broad range of diseases, encompassing various lymphoid and B-cell malignancies [95]. In EBV-associated malignancies, the EBV-encoded nuclear antigen 1 (EBNA1) plays a crucial role as a viral genome maintenance protein. It plays a crucial role in genome replication and genome stability during latency in actively dividing cells. It is expressed in all these malignancies and is responsible for ensuring replication and its maintenance during latency in proliferating cells. Studies on EBV have demonstrated that EBNA1 facilitates viral DNA replication by engaging with RNA G4 structures and recruiting the cellular origin replication complex (ORC). Specifically, the LR1 and LR2 domains of EBNA1 preferentially bind to RNA molecules that can form G4 structures [40]. The EBNA1 glycine-alanine repeats (GAr) mRNA contains several clusters of PQS motifs occurring 13 times throughout the repeat sequence, playing a crucial role as cis-regulatory elements influencing mRNA translation. Both CD spectroscopy and UV thermal difference spectra have confirmed the G4-EBNA1’s ability to fold into a parallel G4 structure, highlighting its unique functional significance [39]. The EBNA1 mRNA restricts MHC class-I-restricted CD8^+^ T cell epitope presentation by CD11c^+^ dendritic cells in lymph nodes and hampers the early priming of antigen-specific CD8^+^ T-cells. Nonetheless, when these G4 structures are destabilized by codon modification, there is a notable enhancement in in vivo antigen presentation and the activation of virus-specific T cells [96]. This suggests that manipulating G4 structures could be a potential strategy to improve immune responses against EBV and related infections. In another study, it has been observed that the cellular protein nucleolin (NCL) plays a crucial role in EBV immune evasion by directly interacting with G4s in the GAr-encoding sequence of EBNA1 mRNA. This interaction enables the virus to evade the host’s immune response [41].

#### 3.3.3. Roseoloviruses (Human Herpesvirus-6 or HHV-6)

Human herpesvirus 6A (HHV-6A) and HHV-6B are two closely related DNA viruses in the Betaherpesvirinae subfamily which exhibit distinct biological and epidemiological characteristics despite their high genome sequence similarities. These viruses are able to integrate their genomes into the telomeres of infected cells, which can fold into G4 structures and may play a role in the process of HHV-6A chromosomal integration. Stabilizing these G4 structures with a G4 ligand (BRACO-19) affects the telomerase complex’s ability to elongate telomeres and hinders the integration of HHV-6A into chromosomes [38].

#### 3.3.4. Kaposi’s-Sarcoma-Associated Herpes Virus (KSHV or HHV-8)

Kaposi’s-sarcoma-associated herpesvirus (KSHV), a member of the Herpesviridae family and genus Rhadinovirus, establishes a lifelong latent infection by persisting as an extrachromosomal episome within infected cells. This process is enabled through the binding of latency-associated nuclear antigen (LANA) to the GC-rich terminal repeats (TRs) region of the viral genome, effectively tethering its epigenome to the host chromosome. Upon analyzing the TRs region, researchers identified potential G4 sequences that can form stable parallel G4 structures in both the forward and reverse strands [75]. Stable G4 structures have also been discovered within the mRNA of the KSHV’s LANA protein, a key KSHV-encoded protein expressed during viral latency and persistence. KSHV adopts a strategy to evade immune detection by keeping LANA protein levels below the threshold needed for host immune system recognition yet enough to maintain the viral genome. The presence of stable G4 structures in the LANA mRNA hinders its translation, thereby regulating antigen presentation. This was further demonstrated by treating cells with TMPyP4, a compound that stabilizes G4 structures [76]. A cellular RNA helicase hnRNP A1 has also been identified as a G4-unwinding helicase which plays a role in unfolding the stable secondary structures in LANA mRNA, thereby regulating its translation. Leveraging this interaction between hnRNP A1 and LANA mRNA could offer potential strategies to control KSHV latency [76]. In a comprehensive genome-wide study of herpesvirus genomes, researchers identified G4-forming sequences in the promoter region of the K15 gene of KSHV. This finding supports the formation of a hybrid G4 structure and highlights the significance of the K15 gene, which is involved in signal transduction pathways and is also present in other human herpesviruses [67].

### 3.4. Hepatitis C Virus (HCV)

The Hepatitis C virus (HCV), a member of the Flaviviridae family and Hepacivirus genus, is a positive-sense, single-stranded RNA virus responsible for causing hepatitis C, a liver disease. Through bioinformatic analysis of various HCV genotypes and subtypes, it was discovered that conserved G4-forming sequences are present in both the positive and negative strands of the HCV genome [55,56]. In a study led by Wang et al. in 2016, it was discovered that the core (C) gene of the HCV contains remarkably conserved G-rich sequences. Further investigation using advanced biophysical techniques such as ^1^H NMR and CD analysis revealed that these consensus sequences could fold into highly stable unimolecular parallel G4 RNA structures [55]. In a different study, the G4Hunter algorithm identified a G-rich sequence (nt 110–131) situated in the stem–loop IIy’ domain of the HCV 3’ negative strand as a potential G4-forming sequence. Through biophysical experiments, it was further confirmed that this RNA sequence can indeed fold into a stable intramolecular G4 structure. Since this G4 is located at the 3’-end of the negative strand, where the replication of the (+) strand initiates, the researchers investigated its potential impact on RNA synthesis by the HCV polymerase in vitro. They demonstrated that the formation of this intramolecular G4 hampers in vitro RNA synthesis by the RNA-dependent RNA polymerase (RdRp) [56]. A recent study employed various biochemical techniques, such as fluorescence anisotropy binding, G4 reporter duplex unwinding, and G4 RNA trapping assays to investigate the behavior of purified recombinant full-length NS3 (nonstructural protein 3) of the HCV and its ability to unfold G4 structures. The findings revealed that NS3 is capable of unfolding synthetic guanine-rich substrates, which mimic the conserved G4 structures found in the negative strand of the HCV genome [97]. These results highlight the G4-mediated role of NS3’s helicase domain in the replication of the HCV.

### 3.5. Human Papillomavirus (HPV)

The human papillomavirus (HPV) is a double-stranded circular DNA virus that belongs to the family Papilomaviridae and can cause skin and genital warts and certain cancers. To date, researchers have identified over 120 types of HPV, but only a few of them pose a threat. High-risk HPVs are associated with the development of precancerous lesions and cancer, while low-risk HPVs typically lead to benign warts and lesions. Among these, cervical cancer—ranked as the fourth most common cancer in women—is predominantly linked to high-risk HPV infections. The genome of HPV harbors several PQSs capable of forming highly stable G4s. However, these PQSs are present in only a fraction of the identified HPV types, specifically in 10 out of 120 types, including some high-risk HPVs such as 16, 18, 52, and 58 [42,58,59,60]. Employing several biophysical techniques, researchers have pinpointed G-rich regions capable of forming stable G4s in seven distinct HPV genotypes. These G4s are located in specific regions such as E1 (HPV32), E4 (HPV9), L2 (HPVs 16, 18, and 57), and LCR (HPVs 52 and 58) regions, which play essential roles in transcription, replication, and the production of viral proteins within the HPV life cycle [42,58,60].

### 3.6. Hepatitis B Virus (HBV)

Hepatitis B virus (HBV) is a member of the Hepadnaviridae family, possessing a genome of 3.2 kb and characterized by a partially double-stranded circular DNA structure. HBV infection leads to various liver-related conditions like acute and chronic hepatitis, liver cirrhosis, and hepatocellular carcinoma. Biswas et al. used Quadparser to identify PQSs in a large dataset of full-length HBV genomes (*n* = 5472). Notably, 92% of HBV genotype B sequences contain a well-conserved PQS, while genotypes A and C have PQS in only a minimal proportion of sequences (1.2% and 0.76% respectively). This PQS motif is located 190 base pairs upstream of the preS2/S transcription start site within the preS2/S promoter of HBV genotype B. Various biophysical methods provided additional evidence, confirming the formation of an intramolecular hybrid G4 structure [61]. In a separate study, a distinct G4 structure in the pre-core promoter region of the HBV genome was identified by employing a blend of bioinformatic and biophysical techniques. This G4 structure was found to be conserved across nearly all HBV genotypes [62].

### 3.7. Filoviruses

Filoviruses are characterized by filamentous virions with a negative-sense, single-stranded RNA genome, about 19 kb in length. This virus group includes Ebola (EBOV) and Marburg (MARV) viruses which are among the deadliest human pathogens causing fatal hemorrhagic fever in humans and primates with extremely high mortality rates [52]. Based on the bioinformatic analysis, Wang et al. reveal the existence of a conserved G-rich sequence within the L gene of *Zaire ebolavirus*. This gene is responsible for encoding the viral RNA-dependent RNA polymerase and may have a role in regulating viral replication and transcription. Through biophysical methods, it was confirmed that this sequence can form a dynamic parallel G4 RNA structure [52]. Furthermore, by using induced CD experiments and the DNA fluorescent probe thiazole orange (TO), it was noticed that multiple sequences obtained from the initial viral isolates of EBOV and MARV have the capacity to adopt G4 structures [53]. This finding highlights the presence of G4s in the genetic material of these deadly viruses.

### 3.8. Zika Virus (ZIKV)

The Zika virus belongs to the Flavivirus genus within the Flaviviridae viral family and is primarily spread to humans through mosquito bites. Using algorithms like QGRS Mapper, G4Hunter, and quadparser, Fleming et al. identified 64–78 PQSs within the ZIKV genome. Among these, seven PQSs were identified in the coding regions of prM, E, NS1, NS3, and NS5 (NS5-A, and NS5-B) genes, and these sequences were found to be conserved across 66 viral genomes from the Flaviviridae family [43]. The Flaviviridae family encompasses several viral genomes, such as Dengue virus (DENV), yellow fever virus (YFV), Japanese encephalitis virus (JE), St. Louis encephalitis virus (SLEV), West Nile virus (WNV), tickborne encephalitis virus (TBE), Langat virus (LGTV), Spondweni virus (SPOV), and Donggang virus (DONV) [43]. Subsequently, the alignment of 78 different ZIKV strain genomes revealed a distinct and exclusive PQS located near the 3’-UTR of the ZIKV genome, which is unique to this virus. Additional experiments using ^1^H NMR, CD, and Tm analysis further confirmed that four of the conserved ZIKV sequences (NS3, NS5-A, NS5-B, and the 3’-UTR) exhibited stable, parallel-stranded G4 formation [43]. Recently, by employing more stringent criteria, four novel PQSs have been identified in the ZIKV, specifically in the NS2, NS4B, and NS5 genes of its genome, alongside previously identified PQSs. Through biophysical and biochemical analysis, it was observed that these PQSs in the ZIKV RNA can form parallel G4 structures [57].

### 3.9. Other Viruses

In addition to the mentioned viruses, numerous others have been found to contain pG4 motifs in their genome or viral proteins capable of interacting with G4s. Analysis of the Simian virus 40 (SV40) genome reveals the presence of sequences potentially forming intramolecular G4s, which are believed to be the natural substrates for the G4 helicase activity of large T-antigen (T-ag) [64,65]. A group of researchers identified six GC boxes (GGGCGG) in the non-coding regulatory region (NCRR) of the SV40 viral genome, forming an unusual quadruplex structure with a C-tetrad between two G-tetrads, as revealed by NMR analysis [66]. These GC boxes serve as binding motifs for SP1 and have a significant role in early transcription. Another study identified approximately 18 putative G4s in the inverted terminal repeat region of the Adeno-associated viruses (AAV) genome. Nucleophosmin (NPM1), an abundant nucleolar DNA-binding protein known to enhance AAV infectivity, interacts with these G4s [98]. A comprehensive genome-wide analysis of 77 H1N1 influenza virus genomes (G4-EA-H1N1) revealed the presence of up to 571 PQSs, indicating a widespread occurrence of RNA G4s in the H1N1 influenza viral genome [99]. All these observations underscore the high prevalence of G4s in the genomes of all Baltimore-classified viruses.

## 4. Non-Canonical G-Quadruplex Structures in Viral Genomes

When G-runs fold into G4 structures, they create G-quartets, while short oligonucleotides in between form connecting loops. This diversity in strand polarities, loop variations, and groove dimensions leads to a wide range of G4 structures exhibiting various topologies [100]. Earlier studies revealed that the stability and structural diversity of G4 DNA depends on its loop length and nucleotide compositions [101]. Until now, research has predominantly focused on conventional G4 structures with loop lengths ranging from 1 to 7 nucleotides. Nevertheless, recent structural investigations have uncovered unconventional G4s (or non-canonical G4s) that deviate from the canonical G4 rules, featuring bulges [70,79,102,103], incomplete G-tetrads (G-triad) [104], and hairpin stem-loop secondary structures [100,105,106]. The overlapping of quadruplex-forming sequences and stem–loop sequences leads to the formation of quadruplex–duplex hybrids, showcasing a blend of their distinctive structural features, and may potentially exhibit novel and unique properties. Emerging experimental evidence suggests that non-canonical G4s, including G4s containing long loops, can form stable hairpin (hairpin-G4) structures and play a regulatory role in the genome, as confirmed by in cellulo reporter assays [100].

Viral genomes have also been shown to harbor G4 structures that deviate from the typical canonical G4 rules, featuring bulges, mismatches, and stem–loops [70,105]. Therefore, to improve the accuracy and presence of PQSs in viral genomes, it is advisable to employ prediction algorithms that consider the possibility of G4s folding from imperfect G-runs. Bioconductor’s PQSfinder tool was one of the advanced G4 prediction algorithms capable of detecting non-canonical G4s that contain bulges or mismatches [107]. Various previous studies, based on viral G4s, have confirmed that the G-rich sequences present within the LTR promoter region of HIV-1 can adopt multiple and dynamically interchangeable G4 conformations [44,45,108]. The LTR promoter sequences have been demonstrated to form LTR-II, LTR-III, and LTR-IV, the three main G4 structures, out of which LTR-III is one of the stable G4 structures exhibiting the highest thermal stability in various in vitro experiments [105]. Based on the NMR structure, Butovskaya et al. revealed that LTR-III G4s fold into a hybrid quadruplex–duplex conformation with a (3 + 1) folding pattern for its G-tetrad core and a 12-nucleotide diagonal loop, forming a hairpin. This distinctive quadruplex–duplex hybrid structure exhibits specific characteristics, particularly a quadruplex–duplex junction, making it an appealing target for drug development [105]. Frasson et al. revealed the existence of G4 structures in the immediate early (IE) gene promoters of *Alphaherpesviruses*, including HSV-1, HSV-2, and VZV. These G4 structures are unique, featuring long loops and bulges, differing from canonical G4 structures, thus emphasizing their conservation and functional significance in these viral genomes [70]. A recent genome-wide study led by Nicoletto et al. analyzed over 12,000 viral genomes belonging to 40 different human-infecting arboviruses, revealing the presence and conservation of PQSs in these viruses. These findings unveiled the existence of both canonical and bulged PQSs, where bulged PQSs represent a significant proportion, comprising 17–26% of all predicted PQSs in arboviruses [103]. Therefore, a more thorough examination of G4s with long loops is crucial for a better understanding of the functional relevance of non-canonical G4s in viral genomes.

## 5. Targeting Viral G-Quadruplexes for Therapeutic Interventions

In the ongoing effort to improve research on combating viruses, the problem of viral infections remains a significant threat to global public health, causing considerable morbidity and mortality. The continuous emergence of drug-resistant viral strains, combined with the current absence of tailored vaccines for numerous viruses, underscores the urgent need for novel therapeutic strategies to effectively combat viral diseases. In this effort, the identification of bioinformatically predicted and highly conserved pG4 motifs within viral genomes, capable of forming G4 structures, stands as a beacon of promise [25]. Targeting G4 structures within viral genomes offers a novel approach to antiviral treatment, providing a valuable tool for a better understanding of viral mechanisms. In the following section, we discuss various available techniques for targeting G4s as a therapeutic agent in the battle against viral infections.

### 5.1. G-Quadruplex-Interacting Ligands Targeting Viral G4

Several small molecules that exhibit the remarkable ability to bind and stabilize G4s have already been developed. These ingeniously designed and synthesized G4 ligands mainly consist of porphyrin, acridine, pyridine, fluoroquinolone, and bisquinolinium derivatives. Remarkably, these G4 ligands often share similar molecular structures characterized by the presence of polycyclic planar aromatic rings, which harmoniously engage G4 structures through π–π stacking and electrostatic interactions. These ligands can interact not only through end-stacking on G-quartets but also by binding within the grooves and loops of the G4 structure. Various methods, such as CD spectroscopy, X-ray crystallography, NMR spectroscopy, surface plasmon resonance (SPR), isothermal titration calorimetry (ITC), and Förster resonance energy transfer (FRET) melting assays, have been employed to characterize the interactions between G4 structures and ligands [109].

**Figure 3:**
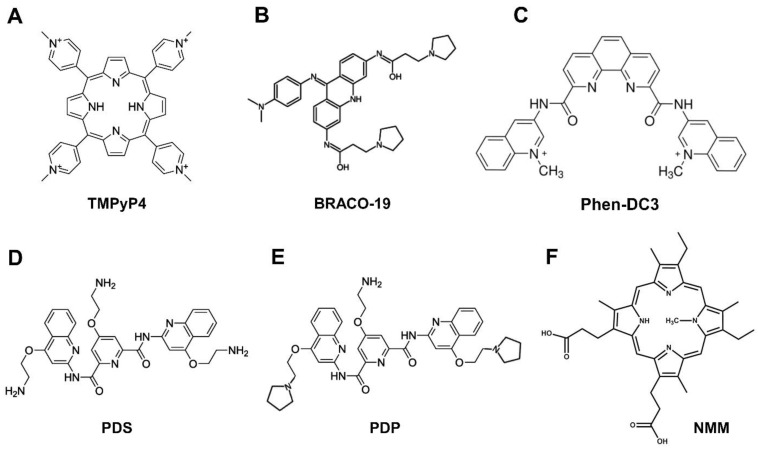
Chemical structures of G4 binding ligands demonstrating antiviral properties. (**A**) TMPyP4; (**B**) BRACO-19 (B19); (**C**) Phen-DC3; (**D**) Pyridostatin (PDS); (**E**) PDP; (**F**) N-methyl mesoporphyrin IX (NMM).

The development of G4 ligands with high specificity for specific G4 structures is crucial for conducting in-depth research into the characteristics and roles of individual G4 structures within the genome. While recent years have witnessed a surge in ligands that can distinguish G4 structures from duplex DNA, the ability to selectively target and study a single, relevant G4 structure amidst many others remains a challenging goal. So far, the most successful approach for selectively targeting G4 structures has primarily relied on distinguishing their topological structures, such as parallel, anti-parallel, and hybrid forms [110,111,112]. Over the past decade, significant progress has been made to improve the binding and specificity of ligands for G4 structures. This has resulted in various chemical classes, displaying strong G4 binding potential, paving the way for potential therapeutic applications against pathogenic microorganisms and viruses. Qian Li and colleagues created G4LDB, a database facilitating the exploration and development of anticancer agents targeting G4 structures. With over 3000 G4 ligands recorded, the majority of these ligands exhibit G4 stabilization properties, while only a limited number can induce G4 destabilization [113]. Exploring stabilizing or destabilizing ligands for multistranded G4 structures has the potential to accelerate drug discovery for previously untreatable viral diseases. Subsequent sections will outline G4 ligands that have thus far demonstrated excellent antiviral efficacy (Figure 3, Table 1).

#### 5.1.1. TMPyP4

TMPyP4, a cationic porphyrin compound, has been extensively investigated as a ligand for binding to G4 structures in antiviral studies (Figure 3A). Through biophysical analysis, it has been observed that TMPyP4 can effectively stack its aromatic core on the upper or lower G4 tetrad and exhibits a slight preference for interacting with quadruplex DNA over duplex DNA. Notably, TMPyP4 demonstrates the ability to stabilize both parallel and antiparallel G4 configurations [114]. Not only does it stabilize G4s, but there are also reports indicating that its interaction with RNA G4s could lead to their destabilization [115]. The availability of TMPyP2, a non-G4-binding structural isomer of TMPyP4, has further propelled the use of porphyrins in G4 research [116]. Overall, TMPyP4 has demonstrated promising antiviral potential across multiple viral strains.

TMPyP4 has been demonstrated to stabilize G4 structures within the HIV-1 *nef* coding region, thereby reducing the Nef protein expression and leading to significant inhibition of Nef-dependent HIV-1 infection in antiviral assays [46]. Furthermore, employing TMPyP4 effectively halted viral replication in two different Jurkat-derived T-cell lines harboring latent HIV-1. This antiviral activity was associated with increased apoptosis or cell death when compared to untreated cells. Notably, this effect was further amplified by synergistically combining TMPyP4 with inhibitors targeting DNA damage repair processes [47]. In the context of the hepatitis C virus, TMPyP4 was responsible for inhibiting C gene expression through the stabilization of viral RNA G4s and subsequently resulted in a dose-dependent reduction in viral RNA levels [55]. TMPyP4 has also been explored to investigate the role of G4s in the EBOV L gene, where it inhibits the transcription of the L gene and EBOV mini-genome replication, suggesting its promise as a potent anti-EBOV agent [52]. In the case of HSV-1, TMPyP4 exhibited the stabilization of the predominant pG4 motifs identified in the repeated regions of the viral genome. While TMPyP4 didn’t impact the entry or replication of HSV-1, it did lead to the confinement of fully infectious HSV-1 virus particles within vesicles [117]. Furthermore, TMPyP4 demonstrated excellent antiviral activity (EC_50_ = 500 nM) against infectious HSV-1 [117]. Within KSHV, TMPyP4 hindered the progression of replication forks, resulting in decreased DNA replication and maintenance of episomes [75]. TMPyP4 also stabilized the G4 structures identified within LANA’s mRNA [76]. Additionally, TMPyP4 has been shown to stabilize G4s formed in the UL2 and UL24 promoters of HHV-1 as well as in the K15 promoter of HHV-8 [67]. TMPyP4 has been further demonstrated to significantly stabilize G4 structures identified within seven different HPV strains (HPV9, HPV16, HPV18, HPV32, HPV52, HPV57, and HPV58) [60]. Among HCMVs, TMPyP4 increased the stability of 36 pG4 motifs identified within 20 viral genes, while preserving their conformation [73]. TMPyP4 was found to effectively hinder the replication and gene expression of SARS-CoV-2 virus RNA G4s in a study by Cui and coworkers [50]. Another investigation focusing on SARS-CoV-2 demonstrated that TMPyP4 displayed potent antiviral activity by targeting SARS-CoV-2 G4s, whereas its positional isomer, TMPyP2, which has a weak affinity for G4, had no impact on SARS-CoV-2 infection. These findings indicate that the antiviral effectiveness of TMPyP4 is mainly due to the stabilization of G4s present in viral genomes [118]. Like SARS-CoV-2, TMPyP4 was also found to effectively hinder the viral growth, genome replication, and protein expression of the ZIKV virus by binding to RNA G-quadruplex structures in a dose-dependent manner [57]. These studies offer a promising approach to combat emerging viral strains. However, TMPyP4’s limited selectivity for G4 structures over duplex DNA raises concerns about potential off-target effects and reduced efficiency of the ligand limiting its biological and clinical application [119].

#### 5.1.2. BRACO-19

The acridine derivative BRACO-19 (B19) is a widely employed G4 ligand recognized for its pronounced preference for G4 structures over duplex DNA and its lower cytotoxicity in comparison to first-generation acridines. These compounds possess a unique structure featuring a central planar pharmacophore that binds to G-tetrads through π–π interactions. Additionally, the two side chains, which are functionalized with tertiary amine moieties, interact with the grooves of G4 structures, enhancing their binding efficacy (Figure 3B) [120]. Among viruses, BRACO-19 was first examined for its effects on the Epstein–Barr virus (EBV), with the aim of understanding the functional and biochemical characteristics of EBNA1. The findings revealed that BRACO-19 effectively hindered EBNA1-dependent stimulation of viral DNA replication and exhibited a preference for inhibiting the proliferation of EBV-positive cells over EBV-negative cells. Additionally, treatment with BRACO-19 disrupted EBNA1’s ability to attach to metaphase chromosomes, implying that its maintenance function is also governed by the recognition of G4s. These results indicate that EBNA1 replication and maintenance rely on the ability of LR1 and LR2 to bind to G4s, offering a potential target for small-molecule inhibitors [40].

Among retroviruses, BRACO-19 exhibits significant anti-HIV-1 activity by stabilizing HIV-1 G4s and impacting various stages of the viral life cycle, including reverse transcription and post-integration processes [48]. Biophysical and biomolecular analysis revealed that the U3 region of the HIV-1 LTR can fold into highly stable G4 structures, which are further stabilized by BRACO-19, effectively hindering HIV-1 reverse transcription [45,48]. The antiviral potential of BRACO-19 was further confirmed in latent HIV-1-infected cells, where it substantially decreased the viral titer to an undetectable level [47]. A comprehensive computational analysis of the HIV-1 *nef* coding region revealed the presence of three contiguous pG4 motifs. BRACO-19 was shown to inhibit Nef expression by stabilizing these pG4 motifs, thereby significantly suppressing Nef-dependent enhancement of HIV-1 infectivity [46]. Apart from that, BRACO-19 also exhibited partial ability to counteract the G4 unfolding activity of NCp7. Therefore, the G4-mediated inhibition of HIV-1 by BRACO-19 at the RNA level arises from both, the stalling of reverse transcriptase and the inhibition of the G4 unfolding activity of NCp7 [84].

BRACO-19 also demonstrated its effectiveness in stabilizing G4 structures within the HSV-1 genome, where multiple pG4 motifs have been identified. It effectively inhibited DNA polymerase processing at these G4 sites, leading to a reduction in the quantity of intracellular viral DNA within infected cells [36]. BRACO-19 was also employed in cells infected with HHV-6A to assess the ability of G4 ligands to hinder viral integration within the telomeric region by enhancing the stability of telomeric G4s. Notably, in cell lines expressing telomerase, treatment with BRACO-19 resulted in a significant reduction in the frequency of chromosomal integration [38]. Furthermore, BRACO-19 has also been shown to stabilize G4s in the promoters of HHV-1 (UL2, UL24) and HHV-8 (K15) genes, indicating a G4-mediated transcriptional regulation within human herpesvirus genomes [67]. BRACO-19 also stabilized the G4s identified within preS2/S gene promoter of HBV genotype B and enhanced the promoter activity [61]. BRACO-19 also demonstrated its ability to effectively suppress ZIKV replication and limit its ability to produce viral proteins, thereby inhibiting the production of infectious particles within ZIKV-infected cells. Overall, these results highlight a promising novel approach for managing and combating ZIKV infections [57]. While BRACO-19 exhibits potent G4 binding abilities and good solubility in water, its limited permeability across biological barriers hinders its pharmacological application. Therefore, it is imperative to develop effective delivery formulations of BRACO-19 in order to explore this novel therapeutic approach.

#### 5.1.3. PhenDC3

Bisquinolinium compounds possess an aromatic core with two protonated quinoline moieties and are able to adopt an intramolecular *syn–syn* hydrogen bond conformation (Figure 3C). PhenDC3, a prominent member of this class, is a highly selective G4 ligand known for its ability to bind to and stabilize G4 structures. PhenDC3 demonstrated its capability to selectively bind and stabilize G4 structures within various HPV genomes leading to consequential impacts on viral transcription, replication, and protein production [60]. PhenDC3 was employed in KSHV to assess its ability to hinder latent viral replication. In KSHV-infected cells, PhenDC3 induced a stress response and hindered DNA replication and episomal maintenance by stabilizing G4s [75]. In Epstein–Barr virus, PhenDC3 has been shown to disrupt the binding between the cellular protein nucleolin and EBNA1 mRNA G4. This blockage of binding leads to the reversal of the GAr-mediated inhibition process of EBNA1 expression and antigen presentation [41]. PhenDC3 was also shown to inhibit HCV replication by binding to RNA G4s at the 3’ end of the intermediate strand [56]. SARS-CoV-2 Nsp3 protein contains a SARS-Unique Domain (SUD), known for its interaction with G4 structures, which is crucial for viral replication. PhenDC3 has demonstrated an inhibitory effect on the SUD/G4 interaction with an IC_50_ of 51 nM [121]. Furthermore, PhenDC3 demonstrated its capacity to stabilize the pG4 motifs identified within the HBV genome [63].

#### 5.1.4. Pyridostatin and Derivatives

Pyridostatin (PDS) is a bisquinolinium derivative with a planar, electron-rich aromatic surface capable of forming hydrogen bonds, enabling it to adapt to the dynamic structures of G4s through its flexible rotatable bonds (Figure 3D). PDS was employed to investigate the role of G4s in EBNA1 mRNA. PDS increased G4 structure stability, resulting in reduced EBNA1 synthesis, highlighting the importance of G4s in viral translational regulation and immune evasion [39]. Furthermore, PDS was employed to investigate the functional relevance of pG4s within the regulatory regions of miRNAs from KSHV (miR-K12) and HCMV (miR-US33). CD spectroscopy and UV melting studies additionally demonstrated that both sequences can form stable parallel G4 structures, which are further stabilized by PDS. Interestingly, PDS-stabilized G4s enhance miR-K12 promoter activity in KSHV—while they reduce miR-US33 promoter activity in HCMV—in a dose-dependent manner [122]. PDS, like BRACO-19, also stabilizes the pG4 motifs in the preS2/S gene promoter of HBV genotype B, leading to an enhancement in its promoter activity, affirming G4-mediated transcriptional regulation within the HBV [61]. In a recent study by Zou et al., PDS demonstrated a potent antiviral activity against ZIKV infection [123]. PDS effectively bound and stabilized ZIKV RNA G4s in vitro and demonstrated strong antiviral activity, outperforming other G4 stabilizing agents and broad-spectrum antiviral ribavirin, with minimal toxicity. PDS inhibited the post-entry stages of the ZIKV replication cycle hampering viral mRNA replication and protein expression. Additionally, PDS hindered the polyprotein processing activity of ZIKV NS2B-NS3 protease, crucial for viral protein production [123].

Wang et al. demonstrated that another G4-stabilizing ligand, PDP (Figure 3E)—an analog of PDS—stabilizes the G4 structures in HCV RNA. This stabilization led to the inhibition of RNA-dependent RNA synthesis and the downregulation of the expression of the HCV C gene. Additionally, PDP effectively hampers the intracellular replication of various HCV genotypes (1a, 1b, and 2a) at both RNA and protein levels, demonstrating strong antiviral properties [55]. Furthermore, Bian et al. used PDS along with PDP to explore the interaction between HCV core RNA G4 structures and the cellular protein nucleolin. The co-localization of HCV core RNA G4 and nucleolin in Huh7.5.1 cells notably decreased in response to both PDP and PDS treatments [124]. These studies confirmed PDS and PDP as promising multi-target viral inhibitors offering insights for designing novel drugs targeting G4s.

#### 5.1.5. N-Methyl Mesoporphyrin IX (NMM)

N-methyl mesoporphyrin IX (NMM) (Figure 3F) exhibits remarkable specificity for G4s, displaying no noticeable binding for other nucleic acid structures, such as single-stranded DNA, double-stranded DNA, triplex DNA, Z-DNA, duplex RNA, or DNA-RNA hybrids. It exhibits a preference for binding to parallel G4s rather than antiparallel ones. The N-methyl group of NMM fits perfectly within the center of the parallel guanine core, where it precisely aligns with potassium ions resulting in effective π–π stacking [125]. Because of NMM’s exceptional selectivity towards G4s, it has been used in many studies involving viruses. In the context of human cytomegalovirus (HCMV or HHV-5), Ravichandran et al. identified 36 PQSs (referred to as GQs), which were linked to 20 specific genes. The majority of these sequences exhibited stable G4 structures, with 28 of them forming parallel G4s, one forming an antiparallel G4 and four displaying mixed conformations. Notably, in this study, NMM demonstrated a stabilizing effect on parallel G4 structures but did not impact a specific antiparallel G4 structure, GQ18, associated with HCMV. This finding confirms the selective binding of NMM to parallel G4 structures. However, in cell-based reporter assays, NMM treatment significantly suppressed UL76 gene expression, possibly due to its impact on GQ18 in the promoter region. This implies that GQ18 can form a parallel G4 structure within the cellular environment. This observation was further confirmed when GQ18 was found to adopt a parallel G4 structure in a NaCl solution [73]. NMM has also been utilized to study the formation of RNA G4s in the genome of the influenza A virus (IAV). By utilizing techniques such as NMR and native polyacrylamide gel electrophoresis, researchers confirmed that the tested sequences, capable of forming G4s in the IAV genome, indeed adopt a parallel G4 structure [54]. This highlights the role of this highly selective binding preference in analyzing G4s and their folding patterns in many biophysical studies on viruses. Notably, NMM is also represented as a “turn-on” fluorescent probe for detecting quadruplex structures with an excitation wavelength of 393 nm and an emission wavelength of 610 nm. This fluorescence intensifies significantly (up to 60 times) when it specifically binds to G4 structures, particularly in the case of parallel G4 binding. This feature enabled the development of extremely sensitive, and highly specific G4-mediated viral detection techniques, which have been applied to various viral genomes such as HIV-1’s and influenza A (H1N1)’s DNA sequences [126,127].

#### 5.1.6. CX-5461

A first-in-class clinical G4-targeting drug, CX-5461, is an RNA polymerase I (Pol I) inhibitor renowned for its ability to selectively bind and stabilize a broad spectrum of G4 structures. CX-5461 is currently undergoing phase I/II clinical trials to assess its effectiveness in treating advanced cancers associated with BRCA1/2 gene deficiencies, positioning it as one of the most advanced and promising G4 ligands in clinical perspectives [128]. In evaluating the impact of CX-5461 on viruses, Westdorp et al. found that CX-5461 treatment during both the initial and advanced stages of HCMV infection effectively suppressed viral DNA synthesis and the subsequent production of the virus [74].

### 5.2. Targeting G4s with Oligonucleotides

Oligonucleotides, or their analogues, offer an effective alternative for addressing the specificity challenges associated with small molecule-based methods. Given that G4s are composed of oligonucleotide-based structures, their remarkable specificity in recognizing base pairs can be harnessed. Alongside the quartets or tetrads, which are the fixed elements constituting the core of these structures, G4s exhibit unique characteristics in their variable components such as loops and flanking regions, which differentiate these structures from one G4 structure to another. Such components ensure the uniqueness of each quadruplex across the genome, making them valuable targets for oligonucleotide or their analogues-based approaches. Like small-molecule G4 ligands, oligonucleotides also engage with their targets in several ways influencing G4 structures through induction, stabilization, or disruption. The use of antisense oligonucleotides (ASOs) has already been explored in several studies to address the selectivity challenges associated with small molecule-based therapeutics targeting G4 structures [129]. Here, we are focusing only on the use of oligonucleotide-based studies in viral genomes.

Hagihara et al. introduced a novel antisense strategy using guanine-tethered antisense oligonucleotides (g-ASs) that is capable of creating a sequence-specific RNA–DNA hetero-quadruplex structure at the targeted RNA sequence. This oligonucleotide rearrangement approach effectively hindered the reverse transcription for a wide range of RNA sequences, including the RNA genome of HIV-1 [130]. The remarkable ability of g-ASs to hinder reverse transcription, translation, and replication processes offers the potential for the development of innovative antiretroviral gene therapies by inhibiting gene replication and expression. Furthermore, Murat et al. demonstrated that using complementary antisense oligonucleotides to disrupt the G4 motifs in the EBNA1 mRNA stimulates the translation of EBNA1 mRNA and enhances antigen presentation [39]. In this study, Murat et al. synthesized a 21-mer RNA 5′-(UCC UGC CCC UCC UCC UGC UCC)-3′ antisense oligonucleotide (AS2) and its DNA analog (dAS2). They showed that AS2 specifically impacted translation by targeting the G4 region, while the DNA version dAS2 had no effect on a control EBV-encoded nuclear antigen 3 (EBNA3A) mRNA lacking G4 structures [39].

Gamma-modified peptide nucleic acid (γPNA) oligomers, like the oligonucleotides, exhibit high-affinity invasion capability into G4 structures identified within West Nile virus (WNV) genomes, holding potential for combating the virus, which affects around 2500 individuals annually in the US, where no antiviral treatments or vaccines exist. The designed γPNA oligomers effectively targeted the G4 structure formed within the NS5 protein-coding region of the WNV genome, exhibiting exceptional binding affinity in the femtomolar range at physiological temperature [131]. Thus, the utilization of γPNA to target pG4 motifs and other related sequences identified within viral genomes could present a novel strategy for inhibiting viral replication and transcription, representing an innovative antiviral approach with broad applications [131]. While an oligonucleotide analogue construct demonstrated promising outcomes in addressing selectivity concerns associated with small molecule therapeutics, an effective in vivo delivery process would remain a therapeutic challenge.

## 6. Discussion: Challenges and Future Perspectives

The resolution of identified G4 structures [132,133,134] and innovative visualization techniques [14,15,16,17] have validated prior computational predictions and in vitro formation of these structures leading to the discovery of new dimensions in the complex biology of G4s. Their actual existence and functional importance highlight the significance of pG4 motifs in shaping genetic regulatory mechanisms across various organisms including both prokaryotic and eukaryotic cells [3,4,5,6,8,9]. Moreover, these sequences have been identified not only in cellular organisms but also in viruses, underscoring their significance in various biological contexts [19,22,24]. The identification of pG4 motifs and their involvement in various stages of the viral life cycle are increasingly evident across most of the Baltimore group of viruses [19,20,25]. However, a significant challenge lies in confirming the existence of G4s in viral genomes and their mechanism of action. As the current number of identified pG4 motifs surpasses the number of well-characterized viral G4s, many of these bioinformatically identified pG4 motifs await comprehensive in vitro and in vivo characterization. Even though certain viruses lack stable G4 structures—such as mumps, measles, polio, and hantaviruses–it is uncertain whether this absence of G4 occurrence is real or due to insufficient studies. Therefore, it is appropriate to say that this demands further investigation of G4s in the viral genomes [23].

Based on the distinct regulatory roles of G4, diverse strategies for targeted therapies can be devised. G4 structures demonstrate unique advantages compared to other nucleic acids, underscoring their significance and making G4s a prime target. G4s stand out due to their well-defined structures and unique electrostatic properties stemming from the arrangement of four sugar-phosphate backbones and a central core of positively charged ions. Moreover, the terminal quartets of G4s provide an ideal platform for the binding of planar aromatic ligands, and the presence of grooves, bulges, loops, and flanking sequences provides extra sites for precise molecular recognition [23]. Numerous studies have consistently demonstrated the evolutionary conservation of G4 motifs over time, and this high degree of conservation across subspecies underscores their potential biological significance [4,5,10,11]. Given the crucial role of G4s, mutations are likely to affect viral fitness, thus constraining the emergence of resistant strains [19]. Additionally, within living cells, G4 structures are believed to exist in a dynamic equilibrium with duplex DNA structures, which can impact normal cellular processes. Leveraging chemical biology techniques to enhance G4-mediated regulation could offer valuable ways to control the viral life cycles more effectively.

G4 structures are diverse and can form in various conformations depending on the specific nucleotide sequences and environmental conditions. Precisely targeting specific DNA/RNA G4 structures is crucial for inhibiting the growth of the viral life cycle without affecting the host cells. To effectively target these different G4 structures, a large screening method is necessary to identify a wide range of compounds, including G4-binding ligands, oligonucleotides, peptides, and proteins that recognize G4 sequences or structures. This will help to determine which types of ligands are most effective against specific G4 targets. Using cell-based screening for G4 ligands resolves the disparities between in vitro and cellular findings, offering a promising approach to identify highly specific ligands [135]. In addition to various methods, another effective approach for identifying potential G4 ligands involves multistep structure-based virtual screening, such as molecular docking, which has demonstrated its effectiveness in narrowing down candidate compounds [136]. VS10 was chosen using a meticulous cascade selection method that effectively ruled out any potential off-target binding to double-stranded DNA [136,137]. Thus, by employing a combination of virtual screening and cell-based screening approaches, the potential for discovering entirely novel bioactive G4 ligands might be enhanced.

Another rapid and efficient high-throughput method for screening and quantifying interactions between proteins, small molecules, and antibodies with G4 DNA and RNA structures is the utilization of customized DNA microarrays [138]. In this context, a group of researchers has successfully developed a large-scale custom DNA microarray system, allowing them to investigate the binding preferences of these biomolecules with approximately 15,000 to 20,000 potential G4 structures [139,140]. Additionally, microarray-based G4-binding specificity assessment is advantageous, as it eliminates PCR amplification steps, which can be challenging for stable G4 templates and is independent of enrichment/pulldown efficiency, unlike sequencing-based methods that can only detect the pulled-down genomic segments [140]. In summary, the G4 DNA microarray offers a high-throughput, efficient, and impartial method for evaluating the effectiveness of G4-binding compounds against a wide range of G4 DNA or RNA structures in a single experiment.

So far, many ligands capable of binding to and stabilizing the G4 DNA structure have been identified. These ligands typically possess planar aromatic rings for π–π stacking with G-tetrads and positively charged components for making electrostatic interactions with the negatively charged phosphate groups of G4s. The advantages of using small molecules include their independence from introducing agents, minimal ongoing labor, easy handling during live-cell experiments, and their flexibility for structural improvements for various in vitro and in vivo applications [110]. The initial challenge in targeting G4 structures with small molecules was achieving high selectivity over double-stranded DNA. However, as the design of G4-binding ligands improved in distinguishing quadruplexes from dsDNA [141], the focus shifted towards differentiating between the existing topologies of folded G4 structures. Studying high-resolution viral G4 structures through NMR spectroscopy and X-ray crystallography has enabled the design of specific G4 ligands by utilizing loops and grooves to achieve molecular recognition while maintaining strong binding affinity. This resulted in the discovery of selective small molecules with the capability to discern between different G4 structures [110]. While certain ligands show a preference for binding to specific structural conformations, they cannot distinguish between unique sequences and may bind to all similar G4 structures, exhibiting similar topologies. Additionally, the long consecutive G-runs, which exhibit different G4 topologies depending on the involved guanine bases from those in long G-rich sequences of the genome, pose a significant challenge for drug designers. The HIV-1-LTR demonstrates a prominent case of a G-rich sequence, forming several quadruplex structures. These structures feature four distinct and overlapping G4-forming regions, known as LTR-I to IV [45]. Therefore, determining the most relevant G4 structures in a physiological environment is a complex task that needs further exploration. In this context, a few unique approaches have been explored to achieve selective binding to specific G4 structures. In their study, Balasubramanian et al. demonstrated the effective utilization of a series of trisubstituted acridine–peptide conjugates for distinguishing between various G4 structures. The substitution pattern and orientation of the peptide substituents, which can distinctly interact with the loops and grooves of G4s, were attached to an acridine core moiety that precisely targets the planar surface of a G-tetrad [142]. Nguyen et al. introduced another novel strategy that combines duplex and quadruplex binders simultaneously to precisely target specific G4 structures with the sequence specificity of duplex binders and the strong binding affinity of quadruplex binders [143]. Tassinari et al. also introduced a novel approach for selectively targeting the HIV-1 LTR G4 region by utilizing naphthalene diimide (NDI)–peptide nucleic acid (PNA) conjugates. This approach capitalizes on NDI’s interaction with the end quartet of the target G4 structures and PNA’s ability to hybridize with the G4 flanking regions, whether they are upstream or downstream, respectively, of the targeted G4s. The NDI–PNA conjugates demonstrated the capability to selectively target the desired G4 structure within the complex HIV-1 LTR region, which contains overlapping G4s competing for recognition. Nevertheless, the only constraint is to ensure that the PNA conjugation sites on the G4 ligand core do not interfere with the ligand’s G4 binding capacity [112].

Another challenging task involves designing site-specific and efficient G4 binding molecules for pathogenic structures that don’t interfere with the host cell machinery. This is because the genome and transcriptome of the host cell contain many potential alternative binding sites for G4 ligands resulting in numerous off-target sites. These cellular off-target sites, which can even outnumber the actual viral target sites, have the potential to divert G4 ligands away from their intended antiviral target, posing a significant challenge in combating the current and future viral outbreaks. With respect to this, an improvement in the off-target activity of G4 binding ligands can offer selective benefits and exclusive targeting of the actual disease-causing viral DNAs and RNAs [23,110,119]. Another challenge is to understand the physiological and biochemical pathways affected by G4 ligands which are hitherto poorly understood. This lack of understanding is due to the limited biological studies that have only examined the effects of these G4 ligands on a small number of genes, rather than comprehensively at the transcriptome or genome-wide levels. Furthermore, contrary to conventional belief, G4 structures do not consistently act as transcriptional repressors, making it difficult to predict how a G4 ligand will impact the expression of a specific gene. It is also essential to emphasize here that the safety of G4 ligands in animal models with viral infections has not yet been evaluated and requires thorough investigation [23]. Nonetheless, the studies performed so far hold significant promise, as they indicate the potential therapeutic targets in the context of viral infections.

In light of the prevailing challenges, where many discovered G4 ligands lack the ability to distinguish specific sequences, there is a pressing need to explore novel methodologies for targeting G4s in both topology- and sequence-specific manners. One potential solution is the adoption of oligonucleotides or their analogues, which can serve as an alternative approach to address the selectivity limitations associated with small molecule-based strategies. Moreover, the revolutionary CRISPR-Cas9 system has offered unparalleled precision in targeting and editing specific DNA sequences within genomes. Despite the extensive knowledge surrounding CRISPR-Cas9 and its potential for targeted therapies, the effectiveness and constraints of this technique in targeting viral DNA/RNA sequences that can form G4 structures remain underexplored [144]. With the extensive development and utilization of the CRISPR-Cas system in viral diagnostic assays [145], there is an urgent need to harness this site-specific gene editing capability for a comprehensive strategy aimed at targeting G4 sequences within the cellular environment. Nonetheless, a fundamental challenge in utilizing oligonucleotides and CRISPR-Cas9 for therapeutic purposes lies in the complex task of their in vivo delivery. Therefore, further research into improving cellular penetration for these approaches becomes necessary. Despite these challenges, the promising potential of all these approaches in viral infection models justifies continued research efforts.

## Figures and Tables

**Table 1:** Summary of G-quadruplexes identified in viral genomes along with reported G4-binding ligands exhibiting antiviral potential.

Family	Virus	Genome (kb)	Presence of pG4 Motifs	G4-Binding Ligands	References
Retroviridae	HIV-1	ssRNA (+) (9.75 kb)	U3 region of 5’ LTR nef	BRACO-19 TMPyP4 PIPER c-exNDI	[44,45,46,47,48,49]
Coronaviridae	SARS-CoV-2	ssRNA (+) (29.7 kb)	ORF1a (position 13,385), S (position 24,268), ORF3a, Membrane (M), RG-1 of Nucleocapsid (N)	BRACO-19 TMPyP4 PDP	[50,51]
Filoviruses	EBOV	ssRNA (−) (18.9 kb)	L gene	TMPyP4	[52]
	MARV	ssRNA (−) (19 kb)	L gene	TMPyP4	[53]
Orthomyxoviridae	IAV	ssRNA (−) (13.5 kb)	PB1 (7KW), PB2 (1KW, 6KW), PA (9KW), HA (11KW)	NMM	[54]
Flaviviridae	HCV	ssRNA (+) (9.6 kb)	C gene	TMPyP4 Phen-DC3 PDP	[55,56]
	ZIKV	ssRNA (+) (11 kb)	3′-UTR, prM, E, NS1, NS2, NS3, NS4B, NS5 genes	BRACO-19 TMPyP4	[43,57]
Papilomaviridae	HPV	dsDNA (8 kb)	E1 (HPV32), E4 (HPV9) L2 (HPVs 16, 18, and 57) LCR (HPVs 52 and 58)	PhenDC3 C8	[42,58,59,60]
Hepadnaviridae	HBV	dsDNA (3.2 kb)	preS2/S promoter	TMPyP4 Phen-DC3 BRACO-19 PDS	[61,62,63]
Polyomaviridae	SV40	dsDNA (5 kb)	Non-coding regulatory region (NCRR)	PDI	[64,65,66]
Herpesviridae	HSV-1	dsDNA (152 kb)	UL2, UL24 gp054 IE promoters pac1 genes	BRACO-19 TMPyP2 c-exNDI GSA-0932	[36,37,67,68,69,70,71]
	HSV-2	dsDNA (155 kb)	IE promoters	BRACO-19	[70]
	EBV	dsDNA (172 kb)	EBNA1 GAr mRNA	BRACO-19 PDS PhenDC3	[39,40,67,72]
	HCMV	dsDNA (235 kb)	Various genes (RL6, UL6, US30, UL34, UL35, UL37, UL51, UL75, UL76, UL82, UL115, UL135, UL138, UL142, US11, US24, US30, IRS1, TRS1)	NMM TMPyP4 CX-5461	[73,74]
	HHV-6	dsDNA (162 kb)	Repeat region of pac-1 TMRs	BRACO-19	[38,67]
	KSHV	dsDNA (170 kb)	Terminal repeat (TR) region, K15 gene, LANA mRNA	PhenDC3 TMPyP4	[67,75,76]

## Data Availability

Not applicable.
